# Broadband Achromatic Metalens for Tunable Focused Vortex Beam Generation in the Near-Infrared Range

**DOI:** 10.3390/nano13202765

**Published:** 2023-10-15

**Authors:** Lvrong Zhao, Xiaoqiang Jiang, Zhihai Wang, Yuwei Chen, Lu Chen, Bo Gao, Weixing Yu

**Affiliations:** 1 Key Laboratory of Spectral Imaging Technology, Xi’an Institute of Optics and Precision Mechanics, Chinese Academy of Sciences, Xi’an 710119, China; zhaolvrong@opt.ac.cn (L.Z.); wangzhihai@opt.ac.cn (Z.W.); chenyuwei@opt.ac.cn (Y.C.); chenlu2022@opt.ac.cn (L.C.); gaobo_101@opt.ac.cn (B.G.); 2 Center of Materials Science and Optoelectronics Engineering, University of Chinese Academy of Sciences, Beijing 100049, China; jiangxiaoqiang@opt.cn

**Keywords:** near-infrared, metalens, broadband achromatic, vortex beam, tunable

## Abstract

Vortex beams accompanied with orbital angular momentum have attracted significant attention in research fields due to their formidable capabilities in various crucial applications. However, conventional devices for generating vortex beams still suffer from bulky sizes, high cost, and confined performances. Metalens, as an advanced platform to arbitrarily control the optical waves, has promising prospects to address the predicament for conventional devices. Although great progress has been demonstrated in the applications of vortex beams, they are still confronted with fixed functionality after fabrication that severely hinders their application range. In this work, the phase-change material of Ge_2_Sb_2_Te_5_ (GST) is employed to design the meta-atoms to realize tunable optical responses. Moreover, the focused vortex beam can be accomplished by superimposing a helical phase and hyperbolic phase, and the chromatic aberrations in near-infrared (NIR) range can be corrected by introducing an additional phase compensation. And the design strategy is validated by two different metalenses (BAMTF-1 and BAMTF-2). The numerical results indicate that the chromatic aberrations for two metalens can be corrected in 1.33–1.60 μm covering the telecom range. Moreover, the average focusing efficiency of BAMTF-1 is 51.4%, and that of BAMTF-2 is 39.9%, indicating the favorable performances of designed BAMTF. More importantly, their average focal lengths have a relative tuning range of 38.82% and 33.17% by altering the crystallization ratio of GST, respectively. This work may provide a significant scheme for on-chip and tunable devices for NIR imaging and communication systems.

## 1. Introduction

As one of the optical vortices, vortex beams possess a toroidal intensity distribution and continuous helical wavefront on account of carried orbital angular momentum (OAM) per photon [1,2]. Specifically, their spiral phase can be identified using the Hilbert factor exp(*lθ*), where *l* and *θ* are the topological charge and angular coordinate, respectively. Benefiting from the unique characteristics as well as the additional degree of freedom to control optical waves, the use of vortex beams has been demonstrated in various practical implantations that may significantly benefit current industrial technologies, for instance, high-dimensional information processing [3,4], super-resolution imaging [5,6], the manipulation of nanoparticles [7,8], etc. Although current techniques for generating vortex beams are quite mature by employing spiral phase plate [9], spatial light modulator [10], and Q-waveplate [11], these conventional optical devices are normally with complex geometrical structures to impart a helical phase that inevitably cause bulk size, high cost and confined performances. Moreover, vortex beams are usually expected to be converged by an additional optical focusing lens for increasing their light intensity. Therefore, these conventional approaches for generating vortex beams are not applicable for miniaturized and integrated optical systems. Metalens, composed of delicately engineered sub-wavelength-scale meta-atoms following certain electromagnetic orders, has promising prospects to address these constraints [12,13,14,15]. Due to its formidable capabilities for locally manipulating the features of optical waves, metalens is competent at providing a compact and efficient platform to further facilitate the current optical systems [16,17].

By employing a Pancharatnam–Berry (PB) phase or propagation phase to design the meta-atoms, the spiral phase profile can be readily imparted to the electric-field of incidence and the focusing phase is also able to be superposed simultaneously. Therefore, the generation of a focused vortex beam (FVB) has been widely investigated by utilizing a single-layer metalens recently. Tang et al. proposed a reflection-type metalens to generate FVB in the near-infrared (NIR) range following PB phase design [18]. Hu et al. designed cylindrical meta-atoms to compose a polarization-independent metalens to generate FVB [3]. Considering the demands in practical scenario, there is no doubt that the FVB generator with a broadband operating range and the ability to correct the chromatic aberration is more desirable due to its attractive functionalities. Ou et al. adopted a polarization-controlled strategy to design a switchable and achromatic metalens to generate FVB from 3.50 μm to 4.75 μm [19]. Song et al. proposed a broadband FVB generator consisting of anisotropic meta-atoms in a long-wavelength infrared range [20]. Although remarkable progresses have been conducted so far, these FVB generators are still confronted with fixed functionality after fabrication, which severely hinders their application range. It is worth noting that several functional materials, including germanium antimony telluride (Ge_2_Sb_2_Te_5_, GST), vanadium dioxide, and indium antimonide, have been employed to design tunable metalens [21,22,23]. However, the metalens for generating FVB with integrated capabilities of broadband operation and tunability is still a challenge that needs to be further investigated.

In this article, a broadband achromatic metalens for tunable FVB generation (BAMTF) is proposed, and it is operating in NIR range that has great significances in substance identification, spectral imaging, space laser communication, etc. The phase-change (PCM) material of GST, which has a noteworthy variation range of its refractive index and moderate optical losses in NIR range, is employed to delicately engineered meta-atoms, offering sufficient phase retardation along with a relatively high efficiency. The PB phase and propagation phase are simultaneously introduced in designing BAMTF to accomplish the FVB generation and eliminate the chromatic aberration in the broadband range. As a demonstration, two BAMTFs with the same operation range (1.33–1.60 μm) covering the telecom range and different parameters are constructed, which are called BAMTF-1 (*l*_1_ = 0) and BAMTF-2 (*l*_2_ = 2). The numerical results indicate that the chromatic aberrations for two BAMTFs are perfectly corrected with maximum deviations of focal length less than 4.8% and 7.0%, respectively. Moreover, the average focusing efficiency of BAMTF-1 is 51.43%, and that of BAMTF-2 is 39.9%, indicating the favorable performances of the designed BAMTF. More importantly, their average focal lengths have the relatively tunable range of 38.82% and 33.17%, respectively. This work may promote the further development of a tunable metalens, and provide a significant scheme for NIR imaging and communication systems.

## 2. Design Principles for BAMTF

### 2.1. Dielectric Constant of GST

Due to the reversible phase change between the amorphous and crystalline states in addition to the excellent opto-electronic characteristics of GST, it was employed to design a metalens for accomplishing tunable FVB generation. In order to identify the performances of the designed metalens, the dielectric constant of GST in NIR range should be investigated to start. In general, the dielectric constant of GST *ε*_eff_(*λ*) during the phase transition can be approximately described by the Lorentz–Lorenz model [24,25]:

(1) $$\frac{\varepsilon_{\mathrm{eff}}(\lambda )-1}{\varepsilon_{\mathrm{eff}}(\lambda )+2}=m\cdot \frac{\varepsilon_{\mathrm{c-GST}}(\lambda )-1}{\varepsilon_{\mathrm{c-GST}}(\lambda )+2}+(1-m)\cdot \frac{\varepsilon_{\mathrm{a-GST}}(\lambda )-1}{\varepsilon_{\mathrm{a-GST}}(\lambda )+2}$$

where *ε*_c-GST_ (*λ*) and *ε*_a-GST_ (*λ*) represent the dielectric constant of GST at crystalline (*m* = 1) and amorphous (*m* = 0) states, respectively, and they can be acquired from spectroscopic ellipsometry experiment by Karvounis et al. [26]. Moreover, *λ* implies the working wavelength, and *m* indicates the crystallization ratio, which can be tuned from *m* = 0 to *m* = 1 by an external stimulus such as a heating stage, ultrafast laser, or electrical switching [21].

**Figure 1:**
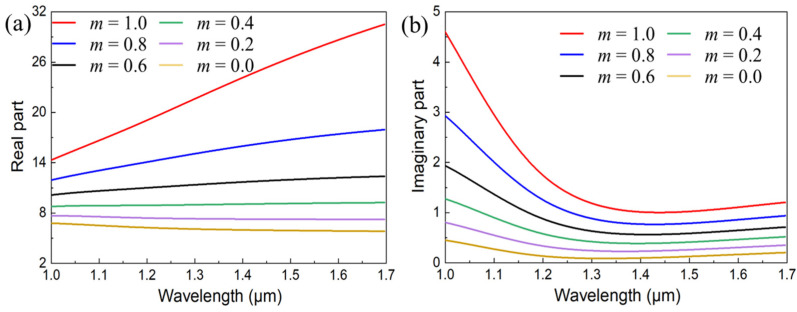
(**a**,**b**) The real and imaginary part of dielectric constants of GST under different crystallization ratios, respectively. Moreover, *m* indicates the crystallization ratio of GST.

Therefore, the real and imaginary part of dielectric constants of GST under different crystallization ratios by steps of 0.2 are plotted in Figure 1. It is clear from Figure 1a that the variation between *m* = 1.0 and *m* = 0.8 was dramatic compared with the others. And such a dramatic variation will cause an adverse impact on the performances of meta-atoms during the tunning processes. In addition, the imaginary part at *m* = 1.0 was also much higher than the others, implying that the optical losses cannot be ignored as they will directly deteriorate the efficiency of BAMTF. Accordingly, the initial state of GST was preset as *m* = 0.8 to design the meta-atoms for the purpose of obtaining relatively high-efficiency and adequate phase compensations.

### 2.2. Broadband Design Principles

Generally, the divergence in transmitting vortex beams is unfavorable in most implementations. Therefore, the generated vortex beams are expected to be converged by additional focusing lens for increasing their light intensity. Due to the powerful functionality, the generation and focusing of vortex beams can be accomplished by a single metalens, which can concurrently impart the helical phase and hyperbolic phase on incident light, and such a phase profile in the *x*-*y* plane can be described as [27,28]

(2) $$\varphi_{1}=-\frac{2\pi }{\lambda }\cdot (\sqrt{x^{2}+y^{2}+f^{2}}-f)+l\cdot \arctan(\frac{y}{x})$$

where *f* represents the preset focal length of BAMTF. However, a conventional focusing metalens is only able to operate at a single wavelength due to the phase discontinuity, and the chromatic dispersion inevitably appears in broadband operation [29]. Therefore, the phase profile should be further modified by introducing an additional phase compensation to correct chromatic aberration in the concerned wavelength range $\lambda \in \left(\lambda_{\min},\lambda_{\max}\right)$, and it is defined as [29,30,31]

(3) $$\varphi_{2}=-[\frac{2\pi }{\lambda }\cdot (\sqrt{x^{2}+y^{2}+f^{2}}-f)]\cdot (\frac{1}{\lambda }-\frac{1}{\lambda_{\max}})$$

where *λ*_min_ and *λ*_max_ are the boundaries of the operating wavelength range, respectively.

The achromatic performances can only be realized when the two parts of the phase demands are simultaneously satisfied. The phase requirement provided by meta-atoms can be separated into a basic phase profile *φ*_1_ and phase compensation *φ*_2_. In particular, the former part is only connected with *λ*_max_ and it can be modulated by introducing a PB phase that rotates each meta-atom to realize NIR-wave focusing. On the contrary, the latter one is the phase difference between *λ*_min_ and *λ*_max_, which is linearly proportional to the operating wavelength. This phase difference will be compensated by exploiting the propagation phase altering the geometric parameters of each meta-atom to the correct chromatic aberration. In addition, the basic phase profile and phase compensation are isolated from each other due to the completely different physical mechanism, which can be separately imparted on the meta-atoms to accomplish achromatic focusing in the concerned wavelength range. Moreover, it should be mentioned that the propagation phase itself has the impact for converging optical waves, and it should be counteracted by the PB phase at *λ*_max_. Otherwise, it may dramatically affect the practical focal length of the designed metalens. Moreover, it should be noted that the method was of a continuous design, indicating that all the chromatic aberration in-between the concerned wavelength range can be corrected.

**Figure 2:**
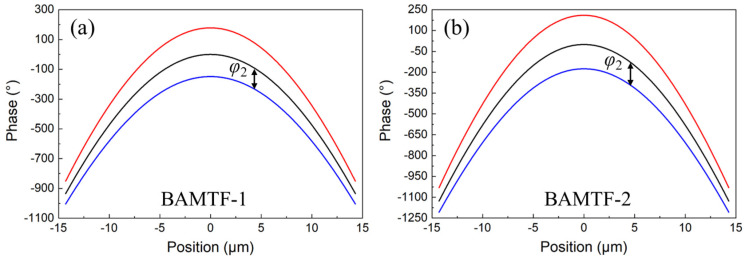
The phase requirements for (**a**) BAMTF-1 and (**b**) BAMTF-2.

Consequently, the phase profiles for engineering BAMTF were the combination of phase requirements in Equations (2) and (3). In this work, the two BAMTFs with the same operation range (1.33–1.60 μm) and different parameters were constructed. In particular, the diameter, focal length, and topological charge for BAMTF-1 were *D*_1_ = 29.15 μm, *f*_1_ = 25.0 μm, and *l*_1_ = 0, respectively. And those of BAMTF-2 were *D*_2_ = 29.15 μm, *f*_2_ = 20.0 μm, and *l*_2_ = 2, respectively. Therefore, according to the aforementioned theoretical analyses, the phase profiles for BAMTF-1 and BAMTF-2 are depicted in Figure 2.

In this work, the preset operating wavelength range was 1.33–1.60 μm, with a relative bandwidth of 18.43%. The operating wavelength range can be increased in our design due to the sufficient phase compensation offered by meta-atoms, specifically, the established meta-atom library (length: 0.300 μm ≤ *a* ≤ 0.505 μm; width: 0.050 μm ≤ *b* ≤ 0.205 μm). However, it is worth to point that the meta-atom associated with the very small value of the phase difference between *λ*_min_ and *λ*_max_ normally possesses a small geometrical size, which will lead to an exceedingly low PCR efficiency. Similarly, the meta-atom associated with the very large value of the phase difference between *λ*_min_ and *λ*_max_ generally has a large geometrical size, which may cause undesired resonances in the concerned wavelength range, that can also deteriorate the PCR efficiency and the linearity of corresponding phase responses. And these defects will deteriorate the focusing efficiency and continuous achromatic performances. Moreover, the preset operation range can cover the telecom range (1.33–1.55 μm), indicating that the proposed BAMTF may provide a significant scheme for NIR communication systems.

### 2.3. The Design of the Metalens

According to the broadband design principles in Section 2.2, both the propagation phase and PB phase are supposed to be introduced by the designed meta-atoms. Specifically, the propagation phase, connected to the effective refractive index of the meta-atoms *n*_eff_, can be modified by altering their geometrical parameters, described as *n*_eff_ · *h* · 2π/*λ* (*h* indicates the structural height). Meanwhile, the PB phase is tightly determined by the rotation angle of meta-atoms under the left circularly polarized (LCP) incidence, and the transmitted electric fields *E_t_* is defined as [32]

(4) $$E_{t}=\frac{t_{o}+t_{e}}{2}|\sigma \rangle +\frac{t_{o}+t_{e}}{2}\exp(\mp i2\sigma \theta )|-\sigma \rangle$$

where *t_o_* and *t_e_* indicate the complex amplitude of the meta-atom along the long and short axes, respectively. Moreover, *σ* and −*σ* are the optical waves with LCP and right circularly polarization (RCP), respectively. And *θ* represents the in-plane rotation angle of the anisotropic meta-atom. Although both LCP and RCP lights exist in the transmission fields, only the orthogonal term can impose an additional phase of 2*θ*. Thus, the designed meta-atoms are expected to be operated as a half-wave plate to increase the polarization conversion ratio (PCR) while introducing the PB phase.

**Figure 3:**
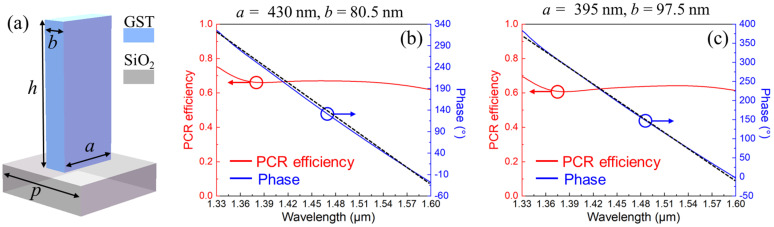
(**a**) Illustration of the designed meta-atom; (**b**,**c**) the PCR efficiencies (red) and phase responses (blue) of the two different meta-atoms, and black dotted-lines indicate the linear fittings.

The illustration of a delicately engineered meta-atom is depicted in Figure 3a. The meta-atom was composed of a GST block on a square silicon dioxide (SiO_2_) substrate. The dielectric constant of GST was taken from Section 2.1. And that of SiO_2_ was acquired from the low-coherence interferometric method of Arosa et al. [33], which has a low dielectric constant and negligible optical losses in the NIR range, making it attractive as a substrate material since it can concentrate the electromagnetic fields in the block with a high dielectric constant to avoid cross-talk between adjacent structures. Moreover, the lattice constant and structural height of designed meta-atom were *p* = 550 nm and *h* = 900 nm, respectively. To investigate the PCR efficiencies and phase responses of the designed meta-atoms, the finite-difference time-domain (FDTD) method was adopted for simulation. Particularly, the conditions of the periodic boundary were applied in the *x*- and *y*-direction, as well as the open conditions being adopted in the *z*-axis. The optical performances of the two meta-atoms with varied geometrical parameters (length *a* and width *b*) are depicted in Figure 3b,c for demonstration.

**Figure 4:**
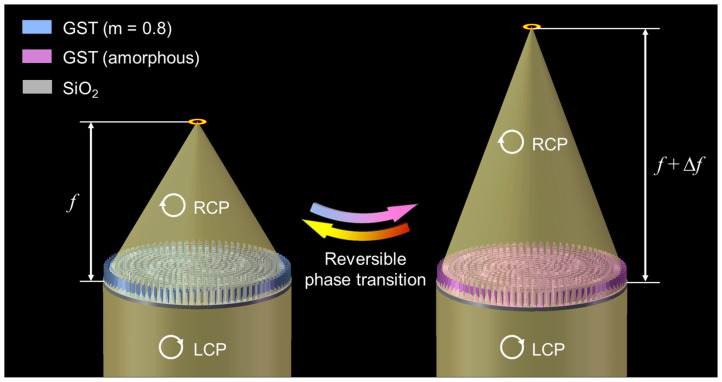
The working principles of the proposed BAMTF, which can generate a focused vortex beam in 1.33–1.60 μm, and their focal lengths can be tuned by altering the crystallization ratio of GST.

It can be observed that the phase responses have great linearity to the operating wavelength, which is extremely critical for correcting chromatic aberrations in the broadband range according to Equation (3). And the linear fittings indicate that the *R*-squared values of the two structures were up to 0.9979 and 0.9977, and the corresponding fitting functions were *y*_1_ = −1306.00*x*_1_ + 2053.96 and *y*_2_ = −1413.03*x*_1_ + 2250.93, respectively. In addition to the great linearity, they also demonstrated relatively attractive PCR efficiencies (>60%). Eventually, according to the design principles and engineered meta-atoms, the BAMTF could be assembled by various meta-atoms following certain phase requirements. The operation principles of BAMTF are shown in Figure 4, and could accomplish focused vortex beam generation in 1.33–1.60 μm. Moreover, the corresponding focal length had a tuning range Δ*f* by altering the crystallization ratio of GST.

As for the practicality of the designed BAMTF, the fabrication methods are described as follows [23,26]. The fabrication of designed BAMTF began with the oxygen cleaning of a SiO_2_ substrate to enhance the adhesion between the substrate and GST film. Then, the GST film was deposited on the SiO_2_ using molecular beam epitaxy. After the electron beam resisted spin-coating on top of the GST film, the meta-atoms following certain orders were patterned using electron beam lithography. Then, a chromium (Cr) film was deposited using E-beam evaporation and could be functioned as a hard mask, and the lift-off could be conducted to remove electron beam resistance. Subsequently, the residual Cr pattern was regarded as an etch mask to transfer the designed pattern onto the GST film using inductively coupled plasma-reactive ion etching. And the remaining Cr mask could be removed by plasma etching.

## 3. The Performances of BAMTF-1

In order to characterize the performances of BAMTF-1, the FDTD method was employed and the conditions of the open boundary were adopted for the three-dimensional cubic of the simulation field, and the LCP waves were incident from the substrate bottom. Furthermore, the electric-field distributions of the transmitted RCP waves, with a wavelength range of 1.33–1.60 μm in steps of 0.045 μm, were observed using far-field construction via Fourier transform. And the achromatic performances and tunable ranges of their focal lengths were investigated as follows.

### 3.1. The Achromatic Performances of BAMTF-1

**Figure 5:**
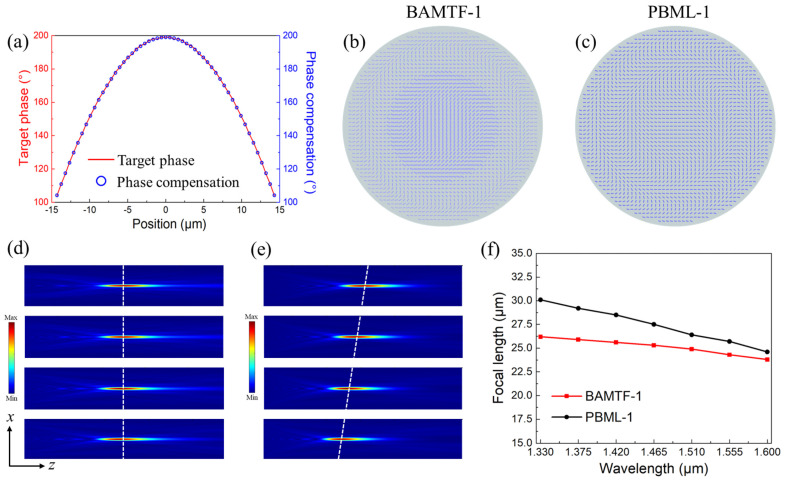
(**a**) The target phase difference (red curve) and realized phase compensation (blue point) of BAMTF-1; (**b**,**c**) the configurations of BAMTF-1 and PBML-1; (**d**,**e**) the electric fields in the transmission plane of BAMTF-1 and PBML-1 at 1.33 μm, 1.42 μm, 1.51 μm, and 1.60 μm, respectively; and (**f**) the corresponding focal length in the operating wavelength of the two metalenses in steps of 0.045 μm.

As for BAMTF-1 (*D*_1_ = 29.15 μm and *f*_1_ = 25.0 μm), which had a topological charge *l* = 0, it could be considered as a special case of vortex beams and had no spiral phase profile, as illustrated in Equation (2). And it was performed as a broadband achromatic metalens. According to the design principles and preset parameters mentioned in Section 2, the BAMTF-1 could be composed of 27 meta-atoms, which had the capability to satisfy the phase difference mentioned in Section 2.2, as depicted in Figure 5a. Furthermore, the comparative structure, only employing the PB phase with a preset operating wavelength of 1.60 μm (the geometrical parameters of selected meta-atom: *a* = 380 nm and *b* = 120 nm), was also calculated for the comparison, which had the same diameter and preset focal length of BAMTF-1 and was named as PBML-1. Their configurations in the *x*-*y* plane are depicted in Figure 5b,c.

The simulated electric fields of the transmitted RCP waves in the *x*-*z* plane are depicted in Figure 5d. It can be clearly observed that the focal lengths, which correspond to the spots possessing the strongest optical power on the transmission path in the *x*-*z* plane, are almost at identical spatial locations in the wavelength range of 1.33–1.60 μm. Meanwhile, although the convergence of the operating RCP waves was accomplished, the chromatic aberration can be clearly observed because its focal lengths were quite different at various operating wavelengths, as depicted in Figure 5e. Their focal spots are plotted in Figure 5f; it can be seen that the average focal length *f*_ave_ of the BAMTF-1 was 25.29 μm, indicating that the actual numerical aperture (NA) was 0.49. And the maximum deviation of the focal length from BAMTF-1 was less than 4.8%, and that of PBML-1 was 20.1%, which further verify that the chromatic aberrations in the operating wavelength can be corrected by the proposed BAMTF-1. In particular, the maximum deviation ratio of the focal length is expressed as 100% × (*f*_m_ − *f*_0_)/*f*_0_, where *f*_0_ is the designed focal length at 1.60 μm, and *f*_m_ indicates the sampled focal length that differs the most from *f*_0_ [20]. 

**Figure 6:**
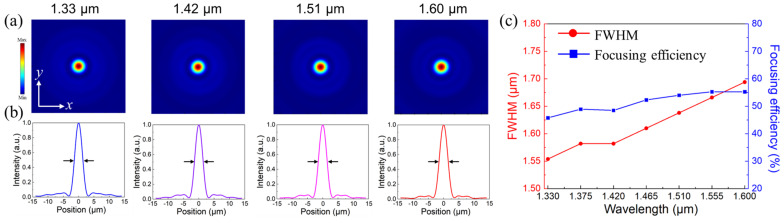
(**a**) The electric fields in the focal plane of BAMTF-1; (**b**) the normalized intensity along the *x*-axis (the black arrows indicate the FWHM); and (**c**) the FWHM (red) and focusing efficiency (blue) of BAMTF-1.

Moreover, the electric-field distributions of the transmitted RCP waves at their focal plane are depicted in Figure 6a. It can be clearly observed that the circular focal spots without diffusions are in the central of the *x*-*z* plane covering the broadband operating wavelength range. Their normalized intensities along the *x*-position exhibited a typical Gaussian-type distribution, as depicted in Figure 6b. And the bright focal spots also demonstrate the attractive functionality of the proposed BAMTF-1. Moreover, the focusing efficiency, one of the most common criteria to evaluate the performances of designed metalenses, was also investigated and defined as the ratio of the optical power from focal spots (with a radius of 3 × FWHM, full width at half maximum) to the incident power [34]. The values of the FWHM extracted from electric field distributions of focal plane were 1.554 μm, 1.582 μm, 1.582 μm, 1.610 μm, 1.638 μm, 1.666 μm, and 1.694 μm, corresponding to the operating wavelength of 1.330 μm, 1.375 μm, 1.420 μm, 1.465 μm, 1.510 μm, 1.555 μm, and 1.600 μm, respectively. And they were all approaching the Abbe diffraction limit [35], implying that the proposed BAMTF-1 has great potential for accomplishing high-resolution imaging. Therefore, the focusing efficiency could be obtained, and it ranged from 45.8% at 1.33 μm to 55.3% at 1.60 μm, with an average focusing efficiency of 51.4%.

As for the focusing efficiency, it is worth noting that the focal spot, with a radius of 3 × FWHM, is one of the most commonly employed definitions [34], and it was adopted here. However, the area of the focal spot is seldom consistently defined. Therefore, the encircled energy metric has been reported to characterize the efficiency of a designed metalens, which calculates the optical power of the focal spots with a radius ranging from 1 × to 10 × FWHM [36], which may provide a brand-new horizon for evaluating the actual performances of designed metalenses.

### 3.2. The Tunable Performances of BAMTF-1

In order to accomplish the tunability of the focal length, the crystallization ratio of GST was altered to change its dielectric constant, which can directly affect the *n*_eff_ of the designed GST meta-atoms to reduce the range of the provided propagation phase that will eventually cause the variation of the corresponding focal length. Therefore, the tunable functionalities of BAMTF-1 were investigated at various crystallization ratios of GST from *m* = 0.8 to *m* = 0 in steps of 0.2 in this section.

**Figure 7:**
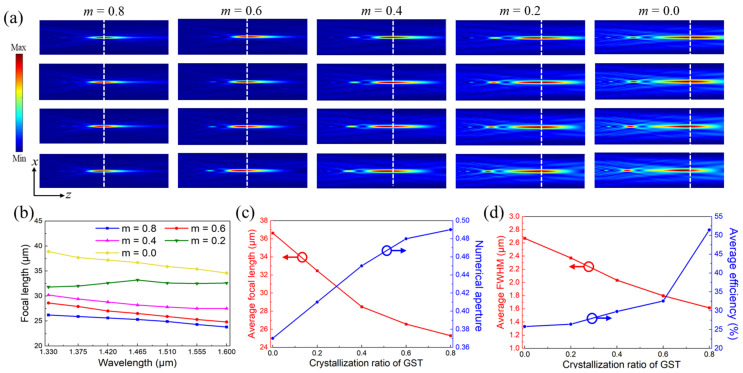
(**a**) The electric fields in the *x*-*z* plane of BAMTF-1 at different crystallization ratios; (**b**) the corresponding focal length in 1.33–1.60 μm in steps of 0.045 μm; (**c**) the average values focal length (red) and NA (blue) at different crystallization ratios; and (**d**) the average values of the FWHM (red) and focusing efficiency (blue) at different crystallization ratios.

It is clear from the electric fields in Figure 7a that the focal length of BAMTF-1 can be tuned at different crystallization ratios of GST due to its various spatial locations. The corresponding focal lengths are plotted in Figure 7b, indicating that the focal length of BAMTF-1 increased as the crystallization ratio of GST decreased. It is worth pointing out that the maximum deviation of the focal length among all the conditions was 4.30 μm (*m* = 0), while that of PBML-1 at *m* = 0 was 6.14 μm, implying that the chromatic aberration can be still corrected to a certain extent during the tunning process. Furthermore, their focal length could be tuned from 23.80 μm (*m* = 0.8 at *λ* = 1.60 μm) to 38.90 μm (*m* = 0 at *λ* = 1.33 μm), corresponding to the numerical aperture (NA) of 0.52 to 0.35, which indicates the relative tuning range of (*f*_max_ − *f*_min_)/*f*_max_ = 38.82% (*f*_max_ and *f*_min_ are the maximum and minimum focal lengths of a metalens under different crystallization ratios of GST, respectively). And their average values are depicted in Figure 7c. However, the average focusing efficiency of the BAMTF-1 clearly decreased during the tuning process (*m* = 0.8 to *m* = 0). Although the GST had ignorable optical losses at a small crystallization ratio, as well as the size of focal spot becoming large, the decreased dielectric constant still had a great impact on its performance due to the appearance of a secondary focal spot and the elongated depth of focus. Moreover, the decreased dielectric constant will cause non-negligible phase divergences that can also deteriorate the focusing efficiency.

## 4. The Performances of BAMTF-2

### 4.1. The Achromatic Performances of BAMTF-2

**Figure 8:**
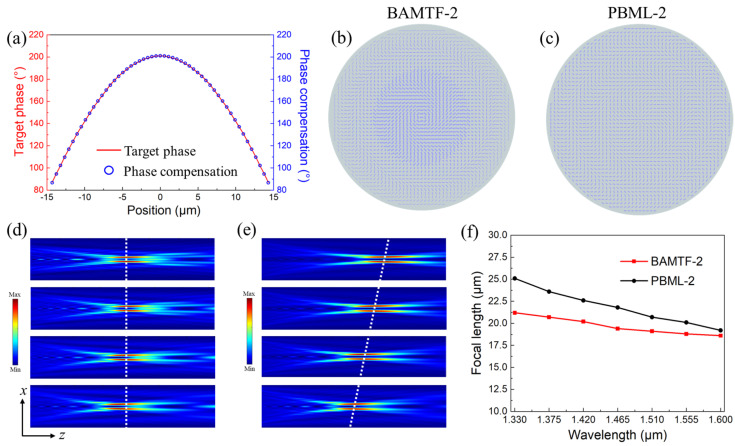
(**a**) The target phase difference (red curve) and realized phase compensation (blue point) of BAMTF-2; (**b**,**c**) the configurations of BAMTF-2 and PBML-2; (**d**,**e**) the electric fields in transmission plane of BAMTF-2 and PBML-2 at 1.33 μm, 1.42 μm, 1.51 μm, and 1.60 μm, respectively; and (**f**) the corresponding focal length in the operating wavelength of the two metalenses by steps of 0.045 μm.

In this section, the same electromagnetic calculation methods and boundary conditions were applied to characterize the designed BAMTF-2 (*D*_2_ = 29.15 μm and *f*_2_ = 20.0 μm) at 1.33–1.60 μm, which was expected to generate the FVB carrying OAM with a topological charge of *l*_2_ = 2. Moreover, the BAMTF-2 consisted of 27 different meta-atoms to perfectly compensate for the phase requirement, as shown in Figure 8a. Similarly, the PB metalens with a preset operating wavelength of 1.60 μm and exactly the same parameters as the BAMTF-2, consisting of identical meta-atoms with *a* = 380 nm and *b* = 120 nm, was also investigated for clear comparison and entitled as PBML-2. Their configurations in the *x*-*y* plane are depicted in Figure 8b,c.

Figure 8d depicts the electric field distributions in the *x*-*z* plane for the proposed BAMTF-2 in the broadband operating wavelength range under the illumination of LCP waves from its substrate bottom. It can be clearly observed that all the sampled focal lengths were nearly constant, indicating that the BAMTF-2 has great achromatic functionality while generating FVB in the concerned wavelength range. As it could be predicted, the chromatic aberration was too conspicuous to be neglected from the electric field distributions from the PBML-2 due to the dramatically varied focal length, as shown in Figure 8e. To elucidate this, their focal spots are plotted in Figure 8f. The acquired *f*_ave_ of BAMTF-2 was 19.71 μm, which corresponded to an actual NA of 0.59; such a high NA is of great importance for practical implementations including microscopy or high-resolution imaging. In addition, the achromatic performances can be also demonstrated by comparing the maximum deviation of the focal length from the BAMTF-2 and PBML-2, which were calculated as 7.0% and 24.5%, respectively.

**Figure 9:**
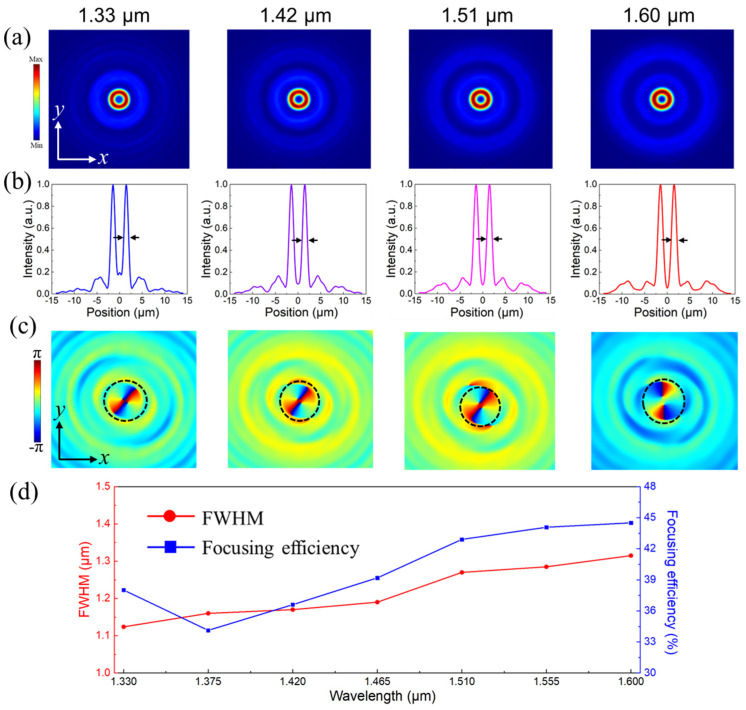
(**a**) The electric fields in the focal plane of BAMTF-2; (**b**) the normalized intensity along the *x*-axis (the black arrows indicate the FWHM); (**c**) the phase profiles of FVB generated by BAMTF-2, and the spiral phase with *l* = 2 is stressed by the black dotted line; and (**d**) the FWHM and focusing efficiency of BAMTF-2.

After the confirmation of the focal length, the electric fields in the focal plane of the proposed BAMTF-2 could be obtained accordingly, and they are shown in Figure 9a. The doughnut-shaped electric fields can be clearly observed in the whole broadband operating wavelength range, implying the existence of FVB with a null-amplitude area at the center of the focal plane, which can also be verified by their normalized intensities along the *x*-position in Figure 9b. Moreover, the phase profiles depicted in Figure 9c indicate that the generated FVB from BAMTF-2 had clear spiral-phase distributions (black dotted-line) in the focal area, which further confirm that the transmitted RCP waves in the range of 1.33–1.60 μm were carrying OAM with a topological charge of *l* = 2. Furthermore, the FWHM and focusing efficiency of BAMTF-2 are plotted in Figure 9d. This is described by the ratio of power inside the doughnut area to that of incident waves [34]. The calculated focusing efficiencies are depicted in Figure 9d by the blue curve, and had an average value of 39.9%.

### 4.2. The Tunable Performances of BAMTF-2

Similarly, the focal length of BAMTF-2 can be also tuned by altering the crystallization ratio of GST due to the variation of phase compensation offered by meta-atoms. The tunable functionalities of BAMTF-2 were investigated at various crystallization ratios of GST from *m* = 0.8 to *m* = 0.2 in steps of 0.2 in this section. It is worth pointing out that the mechanisms of the tunable focal length were exactly the same with BAMTF-1 due to the decreased propagation phase offered by the meta-atoms.

**Figure 10:**
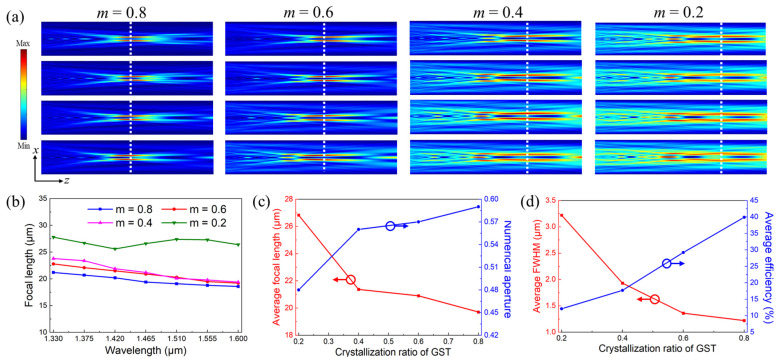
(**a**) The electric fields in the *x*-*z* plane of BAMTF-2 at different crystallization ratios; (**b**) the corresponding focal length in the 1.33–1.60 μm range in steps of 0.045 μm; (**c**) the average values of the focal length (red) and NA (blue) at different crystallization ratios; and (**d**) the average values of FWHM (red) and the focusing efficiency (blue) at different crystallization ratios.

As seen in Figure 10a, the focal length of the BAMTF-2 is able to be tuned corresponding to various crystallization ratios of GST due to the various spatial locations, which can be verified by Figure 10b. Similar to BAMTF-1, the deviation of the focal length from BAMTF-2 will also become conspicuous during the tuning processes (*m* = 0.8 to *m* = 0.2). However, such deviation (4.40 μm at *m* = 0.4) was still less than that of PBML-2, which had the maximum deviation of 5.40 μm at *m* = 0.4, indicating that the chromatic aberration in the concerned wavelength can still be slightly corrected. Moreover, their focal length could be tuned from 18.58 μm (*m* = 0.8 at *λ* = 1.60 μm) to 27.80 μm (*m* = 0.2 at *λ* = 1.33 μm), corresponding to the numerical aperture (NA) of 0.62 to 0.46. And their average values are depicted in Figure 10c. And the relative tuning range of BAMTF-2 was calculated as 33.17% under the crystallization ratio of GST from *m* = 0.8 to *m* = 0.2. However, the averaging focusing efficiency of BAMTF-2 was dramatically decreased due to the secondary focal spot and the elongated depth of focus. The decrease in focusing efficiency was inevitable due to the reduced dielectric constant of GST, which decreases the intensities of interactions between incident light and meta-atoms and eventually deteriorates the PCR efficiency of meta-atoms.

In general, in order to demonstrate the real contribution of our designed BAMTF, the comparison between this work and previously reported works on tunable achromatic metalenses are summarized in Table 1. It can be concluded that achromatic metalenses achieving tunable focal lengths of focused vortex beams are barely investigated, and the proposed BAMTF has the superiorities of the broadband correction of chromatic aberration and a tunable focal length.

Lastly, we want to explain the reasons for selecting the GST in designing the meta-atoms, besides the advantages of a quick response, good stability, and large number of switching cycles [42]. Currently, there are three typical PCMs: vanadium dioxide (VO_2_), GST, and Ge–Sb–Se–Te (GSST). In this work, the designed meta-atoms were a waveguide-like type that required the employed materials to have a relatively large value of dielectric constant. Moreover, we also expected that the dielectric constant of the selected materials could be altered in a wide range under external stimuli in order to accomplish the tunable focal length, which will cause variation in the phase compensation offered by meta-atoms. Therefore, the PCM of VO_2_ is not an ideal candidate in this case due to its relatively low dielectric constant in the insulator state (ε~9). As for GST and GSST, they share similar properties in a large refractive index change during the phase transition. In contrast to GST, the transparency window of GSST can be extended to the long-wave infrared range [43]. However, the development of GST and the processing technique of its micro–nano structure are more mature than that of GSST. In addition, the operating wavelength range in this work was in the NIR range, in which the GST had the clear transparency window. Although there are many different chemical compositions of GST (e.g., GeTe, Ge_2_Sb_2_Te_5_, GeSb_2_Te_4_, GeSb_4_Te_7_, and Sb_2_Te_3_), Ge_2_Sb_2_Te_5_ is still the most commonly employed one due to its relatively longstanding development [44,45,46]. Therefore, Ge_2_Sb_2_Te_5_ was selected to design the meta-atoms in this work.

## 5. Conclusions

In conclusion, two broadband achromatic metalenses made of a GST block on a SiO_2_ substrate were proposed to generate FVB, with topological charges of *l*_1_ = 0 and *l*_2_ = 2, respectively. By simultaneously introducing the PB phase and propagation phase, the delicately designed metalenses, identified as BAMTF-1 and BAMTF-2, were both capable of correcting the chromatic aberration in the broadband operating wavelength range of 1.33–1.60 μm (covering the telecom range), with maximum deviations of the focal length of less than 4.8% and 7.0%, respectively. The numerical results also indicate that the focal length of the two metalenses can both be tuned by altering the crystallization ratio of GST, and their average focal lengths have relative tuning ranges of 38.82% and 33.17%, respectively. The proposed BAMTF may have promising potential for enriching the functionalities of metalenses, which can greatly facilitate the on-chip and tunable devices for NIR imaging and communication systems.

## Figures and Tables

**Table 1:** The comparison between this work and reported tunable achromatic metalenses.

Ref.	Bandwidth	Method	Functionality	Focusing Efficiency
[19]	3.50–4.75 μm	Polarization multiplexing	Switchable topological charge of vortex beam	N/A
[23]	1.8–2.2 THz	Indium antimonide	Tunable focal length from 736.25 to 861.02 μm	13.2–73.3%
[37]	483–620 nm	Polarization switchable	Tunable focal length from 220 to 550 μm	5–50%
[38]	0.9–1.4 THz	Liquid crystal	Achromatic to large dispersion	26.1–33.9%
[39]	440–640 nm	Moiré principle	Continuous zoom range of 1× to 10×	6.06–86.20%
[40]	830–1100 nm	Phase change Material (Sb_2_S_3_)	Switchable operating achromatic band	35–55%
[41]	1310–1550 nm	Alvarez principle	Tunable focal length from 19.8 to 31.2 μm	10–28%
This work	1.33–1.60 μm	Phase change Material (GST)	Tunable focal length from 23.80 to 38.90 μm (BAMTF-1)Tunable focal length from 18.58 to 27.80 μm (BAMTF-2)	26.2–51.4% (BAMTF-1) 11.2–39.9% (BAMTF-2)

## Data Availability

Data will be made available on request.
